# Tract- and gray matter- based spatial statistics show white matter and gray matter microstructural differences in autistic males

**DOI:** 10.3389/fnins.2023.1231719

**Published:** 2023-09-27

**Authors:** Marissa DiPiero, Hassan Cordash, Molly B. Prigge, Carolyn K. King, Jubel Morgan, Jose Guerrero-Gonzalez, Nagesh Adluru, Jace B. King, Nicholas Lange, Erin D. Bigler, Brandon A. Zielinski, Andrew L. Alexander, Janet E. Lainhart, Douglas C. Dean

**Affiliations:** ^1^Neuroscience Training Program, University of Wisconsin-Madison, Madison, WI, United States; ^2^Waisman Center, University of Wisconsin-Madison, Madison, WI, United States; ^3^Department of Radiology and Imaging Sciences, University of Utah, Salt Lake City, UT, United States; ^4^Department of Radiology, University of Wisconsin-Madison, Madison, WI, United States; ^5^Department of Psychiatry, Harvard School of Medicine, Boston, MA, United States; ^6^Department of Neurology, University of Utah, Salt Lake City, UT, United States; ^7^Department of Psychiatry, University of Utah, Salt Lake City, UT, United States; ^8^Department of Psychology and Neuroscience Center, Brigham Young University, Provo, UT, United States; ^9^Department of Neurology, University of California, Davis, Davis, CA, United States; ^10^Department of Pediatrics, University of Utah, Salt Lake City, UT, United States; ^11^Departments of Pediatrics and Neurology, University of Florida, Gainesville, FL, United States; ^12^McKnight Brain Institute, University of Florida, Gainesville, FL, United States; ^13^Department of Psychiatry, University of Wisconsin-Madison, Madison, WI, United States; ^14^Department of Medical Physics, University of Wisconsin-Madison, Madison, WI, United States; ^15^Department of Pediatrics, University of Wisconsin-Madison, Madison, WI, United States

**Keywords:** TBSS, GBSS, autism, microstructure, white matter, gray matter, NODDI

## Abstract

**Background:**

Autism spectrum disorder (ASD) is a neurodevelopmental condition commonly studied in the context of early childhood. As ASD is a life-long condition, understanding the characteristics of brain microstructure from adolescence into adulthood and associations to clinical features is critical for improving outcomes across the lifespan. In the current work, we utilized Tract Based Spatial Statistics (TBSS) and Gray Matter Based Spatial Statistics (GBSS) to examine the white matter (WM) and gray matter (GM) microstructure in neurotypical (NT) and autistic males.

**Methods:**

Multi-shell diffusion MRI was acquired from 78 autistic and 81 NT males (12-to-46-years) and fit to the DTI and NODDI diffusion models. TBSS and GBSS were performed to analyze WM and GM microstructure, respectively. General linear models were used to investigate group and age-related group differences. Within the ASD group, relationships between WM and GM microstructure and measures of autistic symptoms were investigated.

**Results:**

All dMRI measures were significantly associated with age across WM and GM. Significant group differences were observed across WM and GM. No significant age-by-group interactions were detected. Within the ASD group, positive relationships with WM microstructure were observed with ADOS-2 Calibrated Severity Scores.

**Conclusion:**

Using TBSS and GBSS our findings provide new insights into group differences of WM and GM microstructure in autistic males from adolescence into adulthood. Detection of microstructural differences across the lifespan as well as their relationship to the level of autistic symptoms will deepen to our understanding of brain-behavior relationships of ASD and may aid in the improvement of intervention options for autistic adults.

## Introduction

Autism spectrum disorder (ASD) is a heterogeneous neurodevelopmental condition characterized by challenges in social interaction and communication, and repetitive and stereotyped behaviors (American Psychiatric Association, 2013). While ASD is commonly studied in the context of early childhood (Lord et al., 2020; Hirota and King, 2023), ASD is a life-long condition with complex clinical and neurodevelopmental trajectories that continually change across the lifespan and may contribute to challenging behaviors and differences observed in adult life outcomes (Ratto and Mesibov, 2015). Specifically, the neurodevelopmental window from adolescence to middle-adulthood captures a chronically understudied but critical transitionary life period comprised of complex social changes for autistic and non-autistic individuals alike (Elster and Parsi, 2020). However, a lack of knowledge exists surrounding the microstructural brain changes occurring with aging that may support the emergence of behavioral challenges and sub-optimal outcomes in autistic individuals in part due to the high clinical variability and biological heterogeneity that exists within this spectral population. This dearth of knowledge poses major challenges to the development of individualized treatment and support options for autistic individuals after childhood.

Diffusion magnetic resonance imaging (dMRI) probes tissue microstructure by characterizing the random motion of water molecules in biological tissue (Basser and Ozarslan, 2009; Afzali et al., 2021). Diffusion tensor imaging (DTI) quantitatively describes diffusion properties in biological tissue through four scalar measurements: fractional anisotropy (FA), and mean diffusivity (MD), radial diffusivity (RD), and axial diffusivity (AD). While DTI provides quantitative metrics sensitive to the underlying brain microstructure and organization, the complex biological tissue environment of the brain microstructure (e.g., myelin, crossing axons and dendrites) may cause these diffusion patterns to deviate from the assumed Gaussian distribution of the DTI model. Recent dMRI biophysical models aim to model and account for the complexity of the underlying microstructure and provide metrics with increased biological specificity compared to DTI (Alexander et al., 2019). For example, the Neurite Orientation Dispersion and Density Imaging (NODDI) (Zhang et al., 2012) model, were developed to quantify the angular variation of neurites with orientation dispersion index (ODI) and intracellular volume fraction of neurites (FICVF) and aims to account for a wide range of neurite orientation distributions that capture the full spectrum of patterns observed across brain tissue, including the highly disperse dendritic processes of the cortical gray matter microstructure (Zhang et al., 2012). Taken together, dMRI techniques are well equipped to quantitatively describe tissue microstructure in both white matter and gray matter.

Diffusion MRI methods, and in particular DTI, have been widely used to study brain microstructure in ASD (Travers et al., 2012; Conti et al., 2015; Dean et al., 2016; Vogan et al., 2016; Fitzgerald et al., 2019; Thompson et al., 2020; Andrews et al., 2021). Indeed, studies examining white and gray matter microstructural differences in young autistic individuals suggest these differences may be related to alterations in fundamental neurodevelopmental processes (Ouyang et al., 2016; Andrews et al., 2019, 2021; DiPiero et al., 2022). However, neuroimaging studies in autistic adolescents and adults indicate both white matter (Shukla et al., 2011; Langen et al., 2012; Travers et al., 2015; Dean et al., 2016; Wilkinson et al., 2016; Andrews et al., 2019; Fitzgerald et al., 2019; Hattori et al., 2019) and gray matter (Courchesne et al., 2001; Carper et al., 2002; Courchesne et al., 2003; Langen et al., 2012; Zielinski et al., 2014; Gori et al., 2015; Carper et al., 2016; Bletsch et al., 2021; DiPiero et al., 2022) microstructure differences continue to emerge across the lifespan. For example, utilizing DTI to investigate longitudinal white matter microstructure in autistic individuals 3-to-41 years, Travers et al. revealed a differing developmental trajectory of FA in the corpus callosum such that autistic individuals showed decreasing FA with age whereas FA tended to increase with age in non-autistic individuals (Travers et al., 2015). This reduction in FA in autistic individuals in adulthood was also found in a more recent study within the anterior thalamic radiation and right cingulum (Haigh et al., 2020). Age-related differences between diagnostic groups in white matter microstructure have also been reported in autistic adults 30-to-73 years of age, with higher age-related mean MD and RD in ASD within projection and association fiber tracts (Koolschijn et al., 2017). Other DTI studies of adolescents and young adults have described widespread reductions in gray matter FA and increased MD in autistic individuals (Groen et al., 2011; Bletsch et al., 2021). Beyond DTI, advanced diffusion MRI methods have been used to explore white and gray matter microstructure in ASD. For example, studies utilizing NODDI have shown autistic adults to display decreased neurite density (FICVF) in commissural and long-range association tracts (Andica et al., 2021) and increased neurite dispersion (ODI) in in visual brain areas (Matsuoka et al., 2020). Additionally, diffusion kurtosis imaging (DKI) studies of autistic adults have described reduced axial kurtosis (AK) in the body and splenium of the corpus callosum (Hattori et al., 2019) and reduced mean and radial kurtosis (MK, RK, respectively), and MD in autistic compared to neurotypical (NT) males in parietal, frontal, and temporal cortical regions (McKenna et al., 2020). However, more work is needed to further interrogate whole-brain microstructural differences related to ASD, particularly across adolescence to adulthood.

In this cross-sectional study, we utilized Tract Based Spatial Statistics (TBSS) (Smith et al., 2006) and Gray-Matter Based Spatial Statistics (GBSS) (Nazeri et al., 2015, 2017) to examine and characterize whole brain white matter and gray matter microstructure differences between autistic and non-autistic individuals during late adolescence and adulthood. Previous work has widely applied TBSS to understand white matter differences in ASD during adulthood (Gibbard et al., 2013; Hirose et al., 2014; Andica et al., 2021), however, only one study has utilized NODDI in conjunction with TBSS to investigate white matter microstructural differences in ASD, reporting lower neurite density in major white matter tracts of autistic adults (Andica et al., 2021). Further, only one prior study has applied GBSS to investigate cortical gray matter microstructure related to ASD in childhood to early adulthood, reporting decreased neurite density in autistic individuals across widespread gray matter regions and an accelerated increase in neurite density between diagnostic groups (DiPiero et al., 2022). However, to the best of our knowledge, no prior studies have combined TBSS and GBSS to investigate whole brain white matter and gray matter microstructure with NODDI. This study, therefore, addresses critical gaps in the current ASD literature by taking a whole-brain analysis approach with TBSS and GBSS in conjunction with DTI and NODDI to investigate microstructural differences related to ASD into adulthood.

Based upon the extant literature, we hypothesized widespread group mean, and age-related differences in both white matter and gray matter microstructure such that the autistic group would demonstrate decreased neurite density (FICVF) and increased neurite dispersion (ODI) compared to their NT peers. Considering the wealth of previous work investigating microstructural differences in ASD with DTI, we also aimed to expand on previous work with the inclusion of DTI metrics and speculate such differences to be present across DTI metrics in accordance with neuronal disorganization. As ASD is a neurodevelopmental condition, an individual’s abilities and symptoms are not linear across the lifespan; that is, many autistic individuals tend to experience changes in specific symptoms and domains of functioning over their lifetime (Seltzer et al., 2003; McGovern and Sigman, 2005). As such, we additionally investigated relationships between white matter and gray matter microstructure and measures of autistic symptom severity within the autistic cohort. Thus, this study aims to delineate neuroanatomical and microstructural differences present in autistic adolescents and adults and assesses relationships between microstructural organization and autism severity during a critical transitionary life period. Characterizing these neurodevelopmental differences across the lifespan is a critical step in defining the structural nature of developmental differences associated with ASD, as well as in improving and optimizing therapeutic options that can lead to better long-term outcomes for autistic adults.

## Materials and methods

### Participants

Participants consisted of a sample including 78 autistic and 81 NT participants, selected from a broader, existing longitudinal neuroimaging study examining brain development in ASD (Prigge et al., 2021). While participants have completed up to 5 study visits over a 16-year period from 2003–2019, the study’s MRI scanner and neuroimaging protocols were updated in 2017 to include more state-of-the-art imaging techniques. The current study leverages the diffusion MRI data acquired after this upgrade between 2017–2019 and utilizes participant data from the fifth timepoint only.

All participants were male and between the ages of 12 and 47 years at the time of the MRI scan. Participants with ASD were diagnosed based on the Autism Diagnostic Interview-Revised (ADI-R) (Lord et al., 1994), the Autism Diagnostic Observation Schedule (ADOS) (Lord et al., 2000, 2012), DSM-IV (1994) and ICD-10 criteria; all ASD participants in the present study met criteria for a lifetime diagnosis of ASD. Exclusion criteria consisted of a history of severe head injury, seizure disorder, hypoxia-ischemia, genetic disorder associated with ASD (identified with Fragile-X testing or karyotype), known medical cause of ASD diagnosis (e.g., known patient history, and physical exam), and/or other neurological disorders. Neurotypical (NT) participants did not have history of learning, developmental, cognitive, neurological, or neuropsychiatric challenges or conditions. Additional enrollment criteria have been extensively described elsewhere (Alexander et al., 2007a; Zielinski et al., 2014; Lange et al., 2015). Consent was obtained from all adult participants, and both parental consent and participant assent were obtained for participants under the age of 18 years. All study procedures were approved by the Institutional Review Boards at The University of Utah and University of Wisconsin–Madison.

Intelligence (IQ) was assessed at study enrollment and all subsequent timepoints as part of a comprehensive cognitive battery. At the time of their MRI Scan, IQ was measured with the Wechsler Adult Intelligence Scale-Third Edition (Wechsler, 1997) providing indices of Full Scale, Verbal, and Nonverbal IQ. Additionally, the ADOS-2 (Lord et al., 2012) Module 4 was administered to all participants in the ASD group at timepoint 5. A summary of participant demographic information at Timepoint 5 can be found in Table 1.

**Table 1:** Demographic, cognitive, and clinical characteristics of participants.

Sample demographics	NT	ASD	*p* value
*N*	81	78	-
Age (Years); Mean (SD) [Range]	27.04 (6.83) [12.33–46.92]	26.66 (7.28) [14.67–46.41]	0.74
Race			–
Asian	0	0	–
Black	0	1	–
Multi-Racial	3	0	–
White	73	75	–
Not Reported/missing	5	2	–
Clinical Characteristics average score (SD) [Range]			
ADOS	–	6.87 (2.94) [1–10]	–
SRS	–	80.54 (30.89) [19–146]	–
Full Scale IQ	120.37 (10.95) [95–141]	104.72 (18.02) [60–150]	< 0.00001

**ADOS Calibrated Severity Scores *n* = 75 participants; Social Responsiveness Scale (SRS) *n* = 65 participants; Full-scale IQ *n* = 43 NT *n* = 76 ASD.

### Imaging acquisition and processing

Magnetic resonance imaging (MRI) data were acquired at the University Utah on a 3.0 Tesla Siemens Prisma scanner equipped with a 64-channel head coil. Diffusion weighted images (DWIs) were acquired using a multi-shell spin-echo echo-planar pulse sequence. A total of 187 DWIs were acquired, 7 of which were acquired with no diffusion encoding (i.e., b-value = 0 s/mm^2^) and the remaining 180 acquired along non-collinear diffusing encoding directions with b = 350 s/mm^2^ [12 directions], b = 1,000 s/mm^2^ [24 directions], b = 2000 [48 directions], and b = 3,000 [96 directions]. An additional 14 non-diffusion-weighted images with reverse phase-encoded directions were collected for use in correcting susceptibility-induced artifacts. Additional scanning parameters included: repetition time (TR) = 4,870 ms; echo time (TE) = 92.4 ms; flip angle = 78 degrees; multi-band factor = 3; echo spacing = 0.71 ms; bandwidth = 1,654 Hz/Px; 250 × 209 mm field of view; 168 × 140 imaging matrix; 1.5 mm × 1.5 mm in-plane resolution; and 1.5 mm slice thickness. The duration of the diffusion scan was 15 min and 30 s. A T1-weighted structural scan was additionally acquired using an MP2RAGE sequence with the following parameters: TR = 5,000 ms, TE = 2.93 ms, Inversion Times (TI) = 700 and 2030 ms, flip angles = 4° and 5°, field of view = 256 mm, 176 slices, resolution = 1 × 1 × 1 mm.

Following image acquisition, DWIs were processed using an inhouse processing pipeline. Briefly, DWIs underwent Rician noise (Veraart et al., 2016) and Gibbs ringing artifact correction (Kellner et al., 2016) using MRtrix3 (Tournier et al., 2019). The FMRIB software library (FSL) (Jenkinson et al., 2012) was used to correct for susceptibility-induced off-resonance distortions using the pairs of images with reversed phase encoding and *topup* (Andersson et al., 2003); while the eddy tool was used to correct for motion and eddy current-induced distortions (Andersson and Sotiropoulos, 2016). Outlier detection and replacement was enabled to identify and correct for signal dropout artifacts (Andersson et al., 2016) and gradient directions were corrected for rotations (Leemans and Jones, 2009). Bias field correction was performed using the Advanced Normalization Tools (ANTs) N4 algorithm (Tustison et al., 2010) and non-parenchyma signal was removed using FSL’s Brain Extraction Tool (BET) (Smith, 2002). Finally, the entire DWI series was co-registered to the MP2RAGE uniform image using the TiDi-Fused pipeline in order to enhance the apparent spatial resolution (Guerrero-Gonzalez et al., 2022). Specifically, the mean b = 0 image was calculated and spatially aligned to the MP2RAGE uniform image using rigid registration (Jenkinson et al., 2002). The resulting transformation was subsequently applied to the entire DWI series using the (Avants et al., 2011a) and cubic B-spline interpolation, resulting in the diffusion MRI series up-sampled to MP2RAGE resolution. The diffusion encoding directions were additionally corrected for the rotational component of the rigid body transformation. One subject from the ASD group was removed due to motion to reflect the final sample size in Table 1.

Diffusion tensors were estimated at each voxel using a weighted-least squares algorithm as part of the diffusion imaging in python (DIPY) open-source software package (Garyfallidis et al., 2014). Quantitative maps of fractional anisotropy, and mean, radial and axial diffusivity (FA, MD, RD, AD, respectively) were derived (Basser, 1995). DWIs were also fit to the three-compartment Neurite Orientation Dispersion and Density Imaging (NODDI) tissue model (Zhang et al., 2012) in Python using the Diffusion Microstructure Imaging in Python (DMIPY) toolbox (Fick et al., 2019), to provide estimates of the intracellular volume fraction (FICVF), orientation dispersion index (ODI) and isotropic volume fractions (FISO). Quantitative maps were visually inspected for artifacts (i.e., slice intensity banding, FA hyper-intensities, distortions, and/or blurring).

A study specific template was created from each subject’s FA maps using the antsMultivariateTemplateConstruction2.sh script in ANTs (Avants et al., 2011a). DTI and NODDI parameter maps from each subject were then non-linearly warped to the common study-specific template space for subsequent analysis.

### Tract based spatial statistics

**Figure 1:**
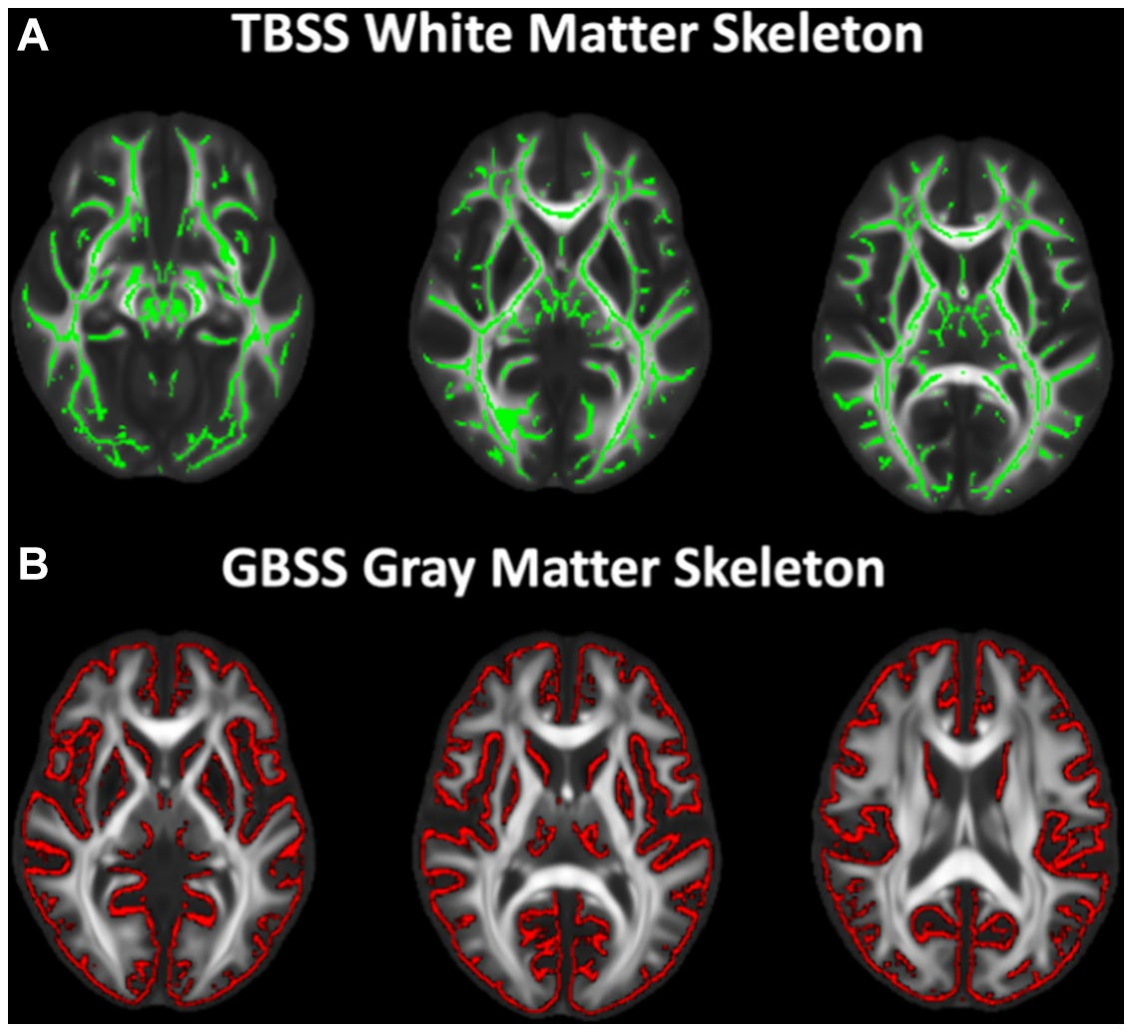
White and gray matter skeletons projected on study-specific template. **(A)** TBSS white matter skeleton (green) generated from skeletonized FA maps representing the center of the white matter tracts. All DTI and NODDI metrics were projected onto the skeleton for voxelwise analysis. **(B)** Gray matter fraction maps were first averaged across subjects and the mean gray matter image was skeletonized (red). All DTI and NODDI metrics and gray matter fraction were projected onto the skeleton from the local gray matter fraction maxima for voxelwise analysis.

Voxelwise analysis of white matter microstructure was performed using Tract-based spatial statistics (TBSS), an analysis method developed to try to alleviate issues with misregistration and data smoothing, and diminish effects of partial volume contamination (Smith et al., 2006). First, a mean FA image was created from all participants and thinned to create a mean FA skeleton representing the center of all tracts common to the population. A threshold of 0.2 was applied to the skeleton to remove voxels of gray matter and CSF resulting in the final TBSS skeleton for analysis (Figure 1A). Each subject’s aligned DTI (FA, MD, RD, and AD) and NODDI (FICVF and ODI) parameter maps were then projected onto the white matter skeleton for voxelwise statistical analysis.

### Gray matter-based spatial statistics

GBSS adopts the TBSS (Smith et al., 2006) framework to enable analyses of diffusion MRI measures in the cortical gray matter (Nazeri et al., 2015, 2017). Processing steps for GBSS have been previously described (Nazeri et al., 2015, 2017). Briefly, a two-tissue class segmentation of the DTI FA maps was performed using *Atropos* (Avants et al., 2011b) to estimate a white matter fraction map. Gray matter fraction maps were then estimated by subtracting the white matter fraction and CSF fraction (NODDI FISO parameter) maps from 1. DTI and NODDI measures and gray matter fraction maps were non-linearly warped into the study-specific template space by applying the warp fields generated by study-specific template construction. Gray matter fraction maps aligned to the study-specific template were averaged to create a mean gray matter fraction map, which was skeletonized using the *tbss_skeleton* tool in FSL (Smith et al., 2006; Jenkinson et al., 2012; Figure 1B). NODDI and DTI metrics were projected onto the gray matter skeleton from local voxels with the greatest gray matter fraction. The gray matter skeleton was thresholded to include only voxels with an average gray matter fraction >0.65 (Nazeri et al., 2017).

### Statistical analyses

#### Microstructural age associations

Age-related associations were first assessed across the TBSS (white matter) and GBSS (gray matter) skeletons separately (Figure 1). First, average values of FICVF, ODI, FA, MD, RD, and AD were extracted from the white matter and gray matter skeletons for each individual without accounting for group. Global mean dMRI values were then used to assess a best fit age model for white and gray matter microstructure trajectory. Linear (dMRI (age) ~ α*age + β) and logarithmic (dMRI(age) ~ α*ln(age) + β) models were fit to the white matter and cortical microstructure measures on a voxelwise basis using RStudio (Version 2021.09.2 + 382 “Ghost Orchid”) (R Core Team, 2021). Information criterion parameters, including the Bayesian Information Criterion (BIC) and Akaike information criterion (AIC), were calculated and used to evaluate the model that best fit the data. The best fitting model for each dMRI parameter across white and gray matter skeletons was subsequently used to investigate population-wise age relationships and differential white matter and cortical microstructural patterns between groups.

#### Group differences and age by group interactions

General linear models (GLMs) were used to investigate group-wise white and gray matter microstructural differences, while age-by-group interactions were used to investigate age-related differences between diagnostic groups. For both TBSS and GBSS analyses, group difference and age-by-group interaction models were run separately. Following the BIC and AIC model fitting for age described above, TBSS white matter analyses utilized logarithmic age in GLMs with measures of FICVF, MD, RD and AD, while linear age was controlled for in GLMs with measures of FA and ODI (Table 2). Likewise, gray matter analyses performed with GBSS utilized logarithmic age in GLMs for measures of FICVF, ODI, MD, RD, and AD, whereas linear age was controlled for in the GLM for FA (Table 3).

**Table 2:** BIC and AIC age fitting across white matter skeleton: ** bolded values indicate best fit according to BIC and AIC metrics.

	FICVF	ODI	FA	MD	RD	AD
BIC: Log Age	**−713.991**	−1,065.134	−797.923	**−3,038.257**	**−3,036.685**	**−2,859.505**
BIC: Linear Age	−712.098	**−1,065.637**	**−799.196**	−3,035.447	−3,035.995	−2,857.291
AIC: Log Age	**−723.198**	−1,074.341	−807.130	**−3,047.464**	**−3,045.892**	**−2,868.712**
AIC: Linear Age	−721.305	**−1,074.844**	**−808.403**	−3,044.654	−3,045.201	−2,866.497

**Table 3:** BIC and AIC age fitting across gray matter skeleton: ** bolded values indicate best fit according to BIC and AIC metrics.

	FICVF	ODI	FA	MD	RD	AD
BIC: Log Age	**−1,015.932**	**−989.927**	−1,206.414	**−3,155.106**	**−3,159.127**	**−3,130.197**
BIC: Linear Age	−1,003.294	−988.118	**−1,207.141**	−3,142.637	−3,148.668	−3,115.656
AIC: Log Age	**−1,025.139**	**−999.133**	−1,215.620	**−3,164.313**	**−3,168.334**	**−3,139.404**
AIC: Linear Age	−1,012.501	−997.325	**−1,216.348**	−3,151.844	−3,157.875	−3,124.863

Non-parametric permutation testing (*n* = 500) was carried out using Permutation Analysis of Linear Models (PALM) (Smith et al., 2006; Winkler et al., 2014). For both TBSS and GBSS analyses, multivariate analyses were performed for dMRI measures that followed the same age-trajectory, whereas a univariate analysis with linear age was run for GBSS analyses of FA. Joint inference of group differences was assessed with Non-Parametric Combination (NPC) and Fisher’s combining function, while differences in individual metrics were also evaluated. Threshold free cluster enhancement (TFCE) (Smith and Nichols, 2009) was used to identify significant regions at *p* < 0.05, FWER-corrected across modality and contrast.

#### Associations with ADOS-2 CSS and SRS

Within the ASD cohort, levels of autistic symptoms were quantified with the ADOS-2 (Lord et al., 2000). The calibrated severity score (ADOS-CSS) is considered a reliable measure of the level of autistic symptoms (Hus and Lord, 2014) and ranges from 1-to-10 (with 10 being the most severe) (Gotham et al., 2009). Non-parametric inference of voxelwise TBSS- and GBSS-skeletonized DTI and NODDI measures were estimated by linear regression using PALM and *n* = 500 permutations, controlling for the effects of age and IQ. Significance was defined as *p* < 0.05, FWER-corrected using TFCE (Smith and Nichols, 2009).

We also investigated relationships between microstructural metrics and parental reported Social Responsiveness Scale (SRS; Constantino and Gruber, 2012) total T-Score. The SRS was developed as a quantitative scale that measures the presence and extent of autistic social impairment. Non-parametric inference of voxelwise TBSS- and GBSS-skeletonized DTI and NODDI measures were estimated by linear regression using PALM with *n* = 500 permutations controlling for the effects of age and IQ. Significance was defined as *p* < 0.05, FWER-corrected using TFCE (Smith and Nichols, 2009).

Significant gray matter regions from GBSS analyses were identified by linearly co-registering statistical maps to the Harvard-Oxford Cortical atlas (Frazier et al., 2005; Desikan et al., 2006; Makris et al., 2006; Goldstein et al., 2007) and the FSL flirt tool (Jenkinson et al., 2012). Similarly, statistical maps from TBSS analyses were registered to the JHU ICBM-DTI-81 (Mori et al., 2005; Wakana et al., 2007; Hua et al., 2008) white-matter labels atlas using the FSL flirt tool (Jenkinson et al., 2012) to identify significant white matter regions.

## Results

### Age relationships across white and gray matter skeletons

**Figure 2:**
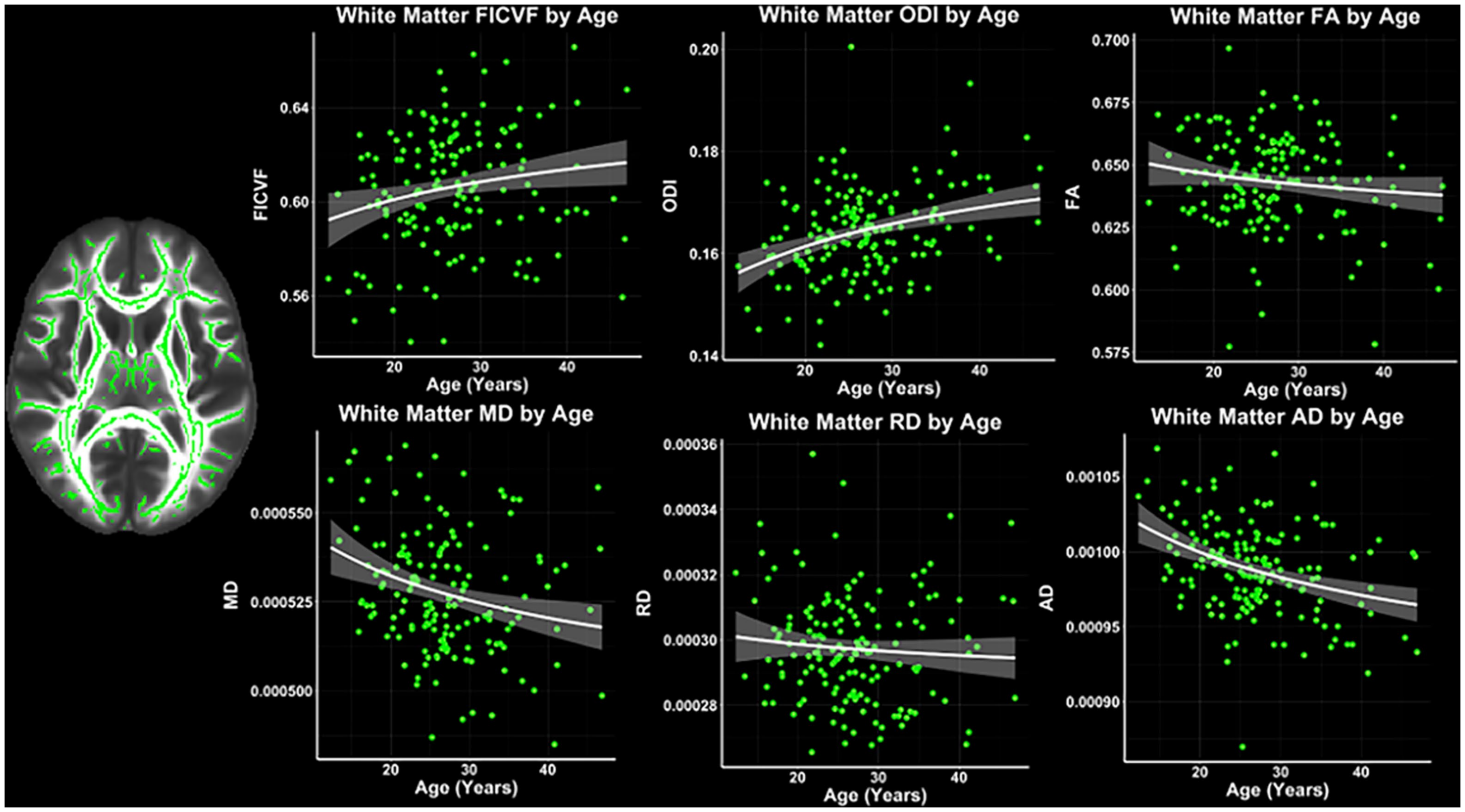
White matter microstructure age relationships from TBSS. Logarithmic and linear fit lines applied per Bayesian and Akaike Information Criterion (BIC and AIC) model selection in Table 2. Scatter points represent mean dMRI measures across the TBSS white matter skeleton shown in Figure 1A and to the left of the scatter plots. Bands represent 95% confidence intervals.

Across the TBSS skeleton, logarithmic growth models were found to best describe the age-related trajectories for FICVF, MD, RD, and AD, while the linear model was more appropriate for FA and ODI. BIC and AIC values from comparison of logarithmic and linear age models for TBSS are provided in Table 2. Average age-related patterns of TBSS-skeletonized diffusion MRI metrics are shown in Figure 2. In general, FICVF and ODI increased with age, while MD, RD, and AD decreased with age. FA tended to remain relatively flat across the investigated age range.

**Figure 3:**
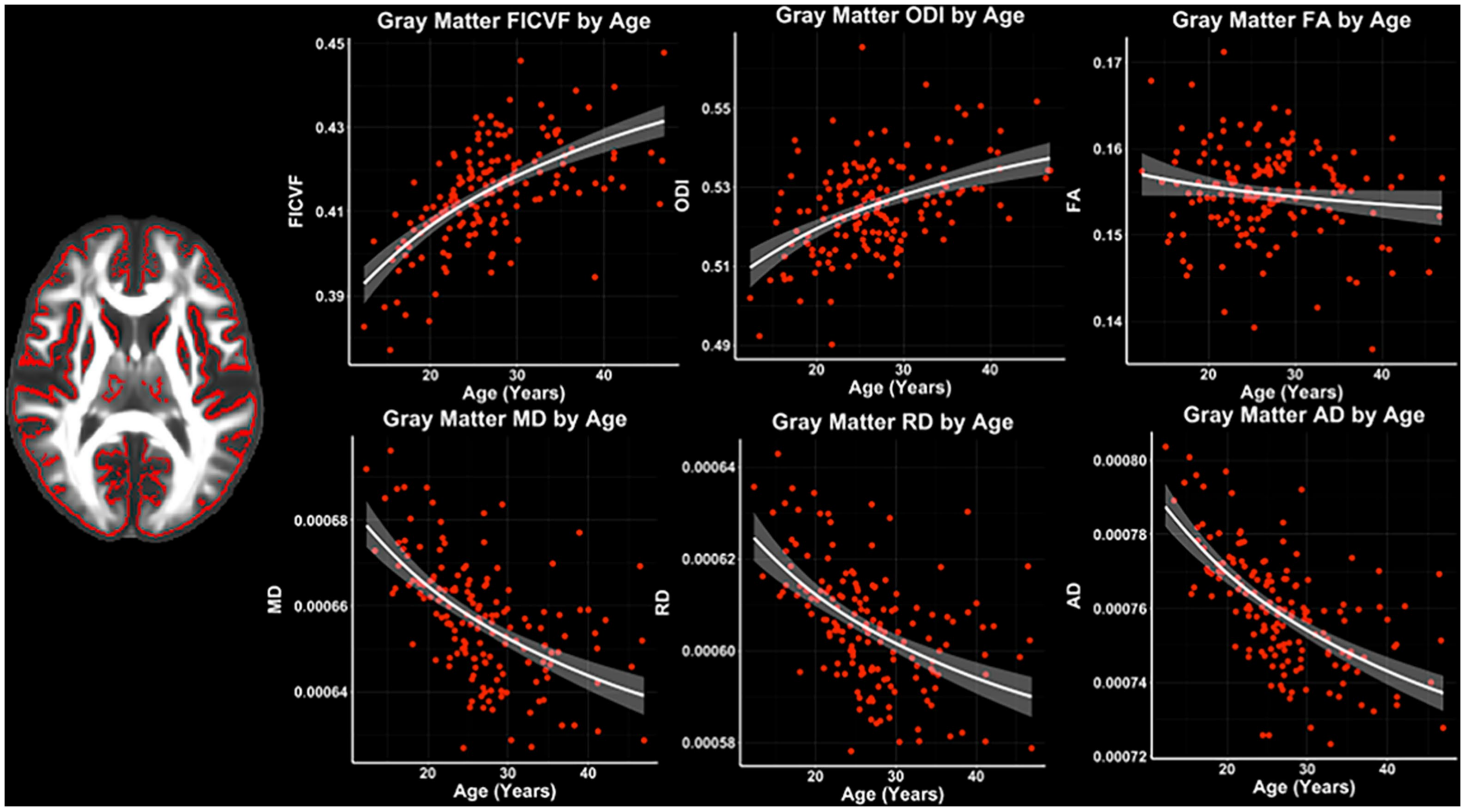
Gray matter microstructure age relationships from GBSS. Logarithmic and linear fit lines applied per Bayesian and Akaike Information Criterion (BIC and AIC) model selection in Table 3. Scatter points represent mean dMRI measures across the GBSS gray matter skeleton shown in Figure 1B and to the left of the scatter plots. Bands represent 95% confidence intervals.

Across the GBSS skeleton, logarithmic growth models best described the age-related trajectories for FICVF, ODI, MD, RD, and AD, while FA was best described by linear-age models. BIC and AIC values from comparison between logarithmic and linear age models for GBSS are provided in Table 3. Average age-related patterns of GBSS-skeletonized diffusion MRI metrics are shown in Figure 3. Generally, FICVF and ODI increased with age, while MD, RD, and AD decreased with age. FA tended to remain relatively flat across ages.

### Microstructural age associations

**Figure 4:**
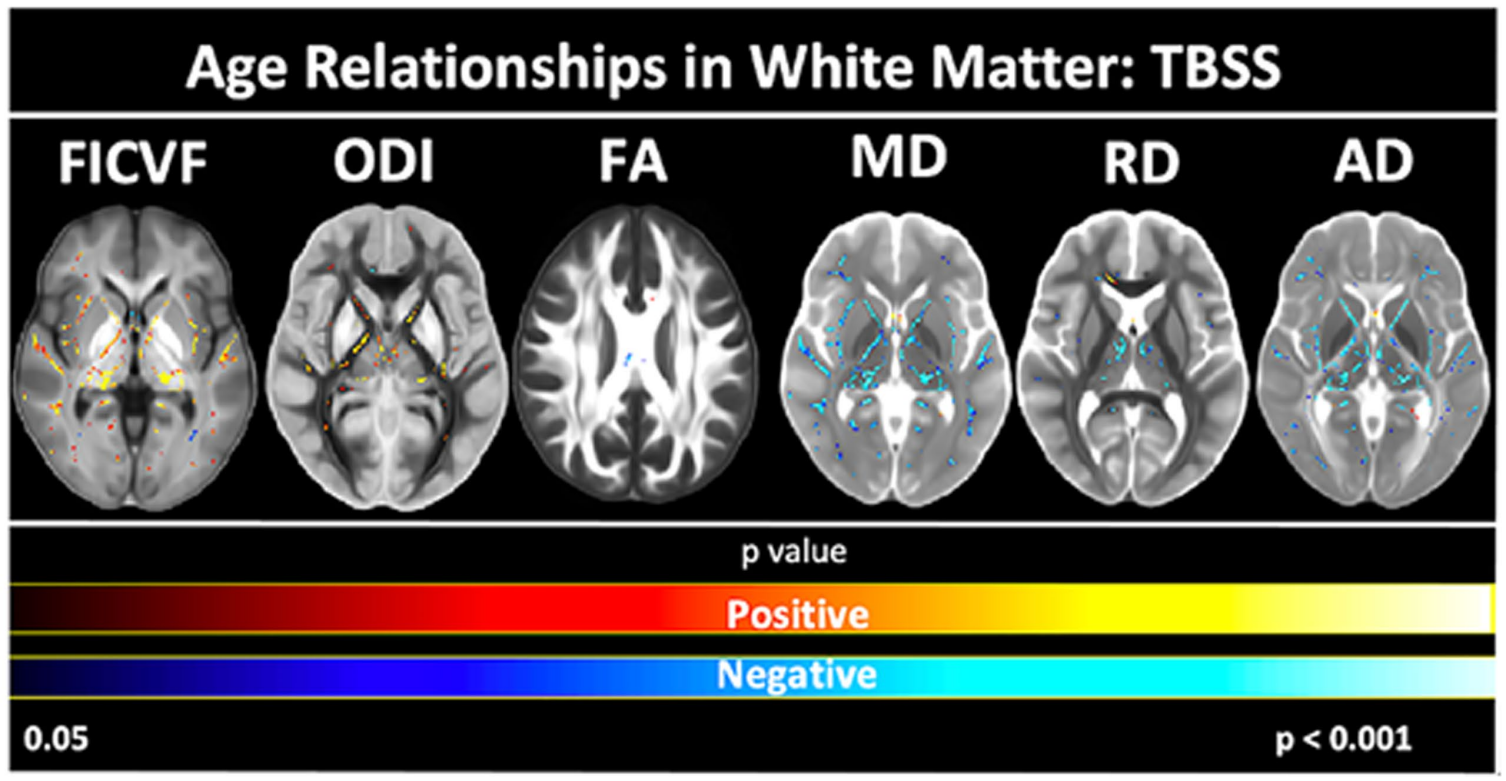
Voxel-based age relationships of white matter microstructure. Logarithmic and linear fits applied per Bayesian and Akaike Information Criterion (BIC and AIC) model selection in Table 2. Significant positive (Yellow/Red) and negative (Light blue/Dark blue) voxels are shown on the dMRI maps for each measure. Color bars represent level of significance.

To further assess the white and gray matter microstructural age relationships, voxelwise TBSS- and GBSS-skeletonized DTI and NODDI measures were fit to logarithmic and linear growth models to assess age-related changes. Across the TBSS skeleton, age-related patterns were generally consistent with the global mean age-related trajectories for each measure described in Figure 2. Measures of FICVF and ODI tended to increase with age (*p* < 0.05; FWER-corrected) across the TBSS skeleton, while measures of MD, RD, and AD tended to decrease with age (*p* < 0.05; FWER-corrected). FA showed sparse areas of decrease. Across white matter measures, relationships with age were consistently noted in white matter tracts including the fornix, anterior and posterior limbs of the internal capsules, external capsules, genu, body, and splenium of the corpus callosum, and anterior corona radiata. Statistical maps for significant age relationships can be visualized in Figure 4.

**Figure 5:**
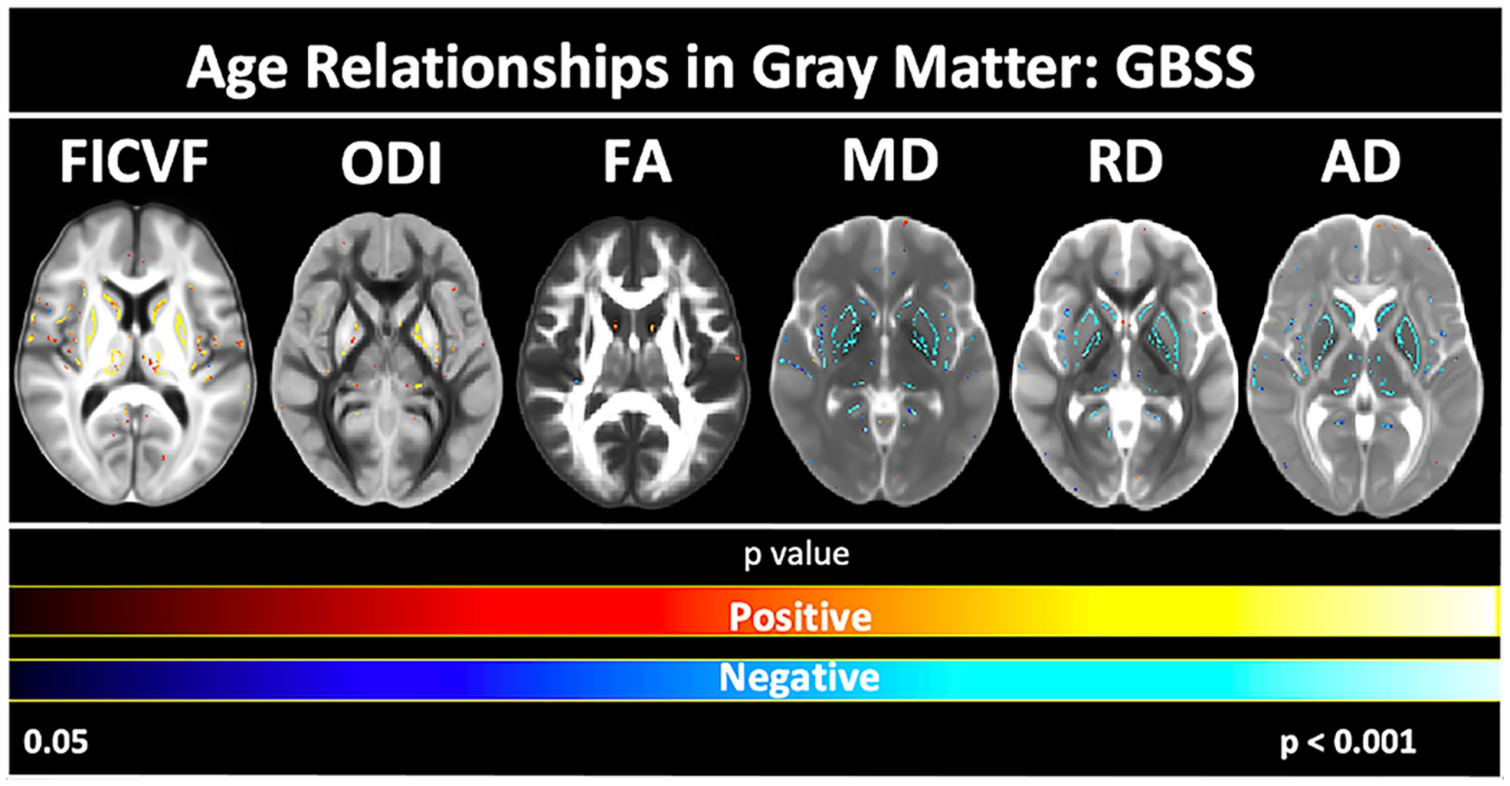
Voxel-based age relationships of gray matter microstructure. Logarithmic and linear fits applied per Bayesian and Akaike Information Criterion (BIC and AIC) model selection in Table 3. Significant positive (Yellow/Red) and negative (Light blue/Dark Blue) voxels are shown on the dMRI maps for each measure. Color bars represent level of significance.

Age-related patterns across the GBSS skeleton were also consistent with the global mean age-related trajectories for each measure described in Figure 3. Measures of FICVF, and ODI tended to increase with age (*p* < 0.05; FWER-corrected) across the GBSS skeleton, while measures of MD, RD, and AD tended to decrease with age (*p* < 0.05; FWER-corrected). FA showed small clusters of significant increases with age. Across gray matter measures, relationships with age were consistently observed in the insular cortex, precentral gyrus, postcentral gyrus, and the central opercular cortex. Statistical maps for significant age relationships can be visualized in Figure 5.

### ASD and NT group differences across white and gray matter microstructure

**Figure 6:**
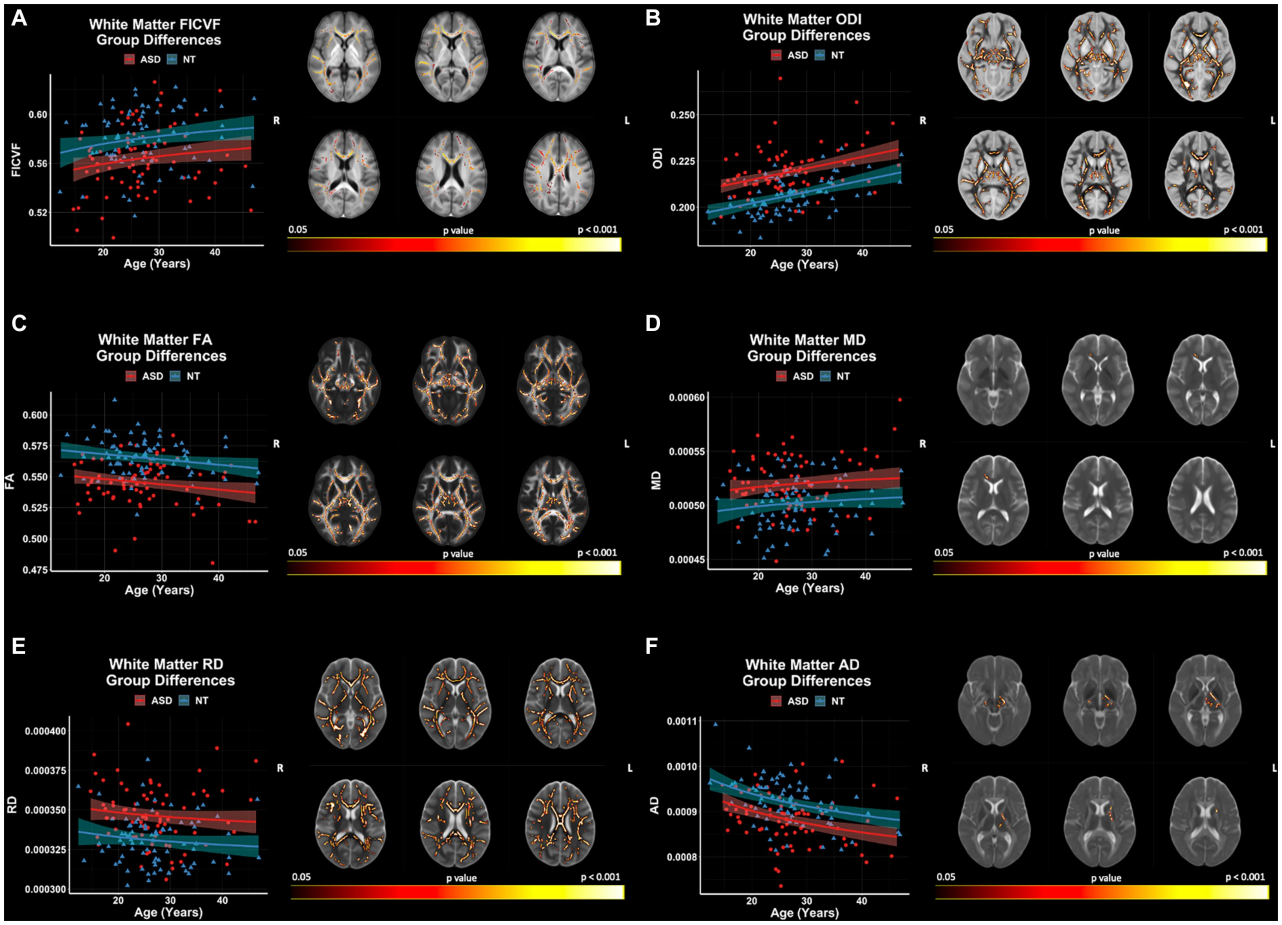
Group differences in white matter microstructure across NODDI and DTI measures. Level of significance and neuroanatomical location of voxels from group difference model are displayed on the mean dMRI maps from all participants. Scatter points (NT = blue; ASD = red) represent mean dMRI values of significant voxels for each measure. Trendlines show model prediction and 95% confidence intervals for group difference on dMRI measures when accounting for the effects of age. **(A)** Significant group difference for FICVF. **(B)** Significant group difference for ODI. **(C)** Significant group difference for FA. **(D)** Significant group difference for MD. **(E)** Significant group difference for RD. **(F)** Significant group difference for AD.

Group comparisons of white matter microstructure using TBSS revealed significant (*p* < 0.05, FWER-corrected) FICVF, ODI, FA, MD, RD, and AD differences between ASD and NT groups (Figure 6). The ASD group demonstrated lower FICVF, and AD, and higher ODI, MD, and RD compared to the NT group across widespread white matter tracts. FICVF, ODI, MD, and RD were all observed to differ between the groups in the anterior corona radiata and much of the corpus callosum. All neuroanatomical locations of significant group differences by dMRI measure can be found in Supplementary Table S1.

**Figure 7:**
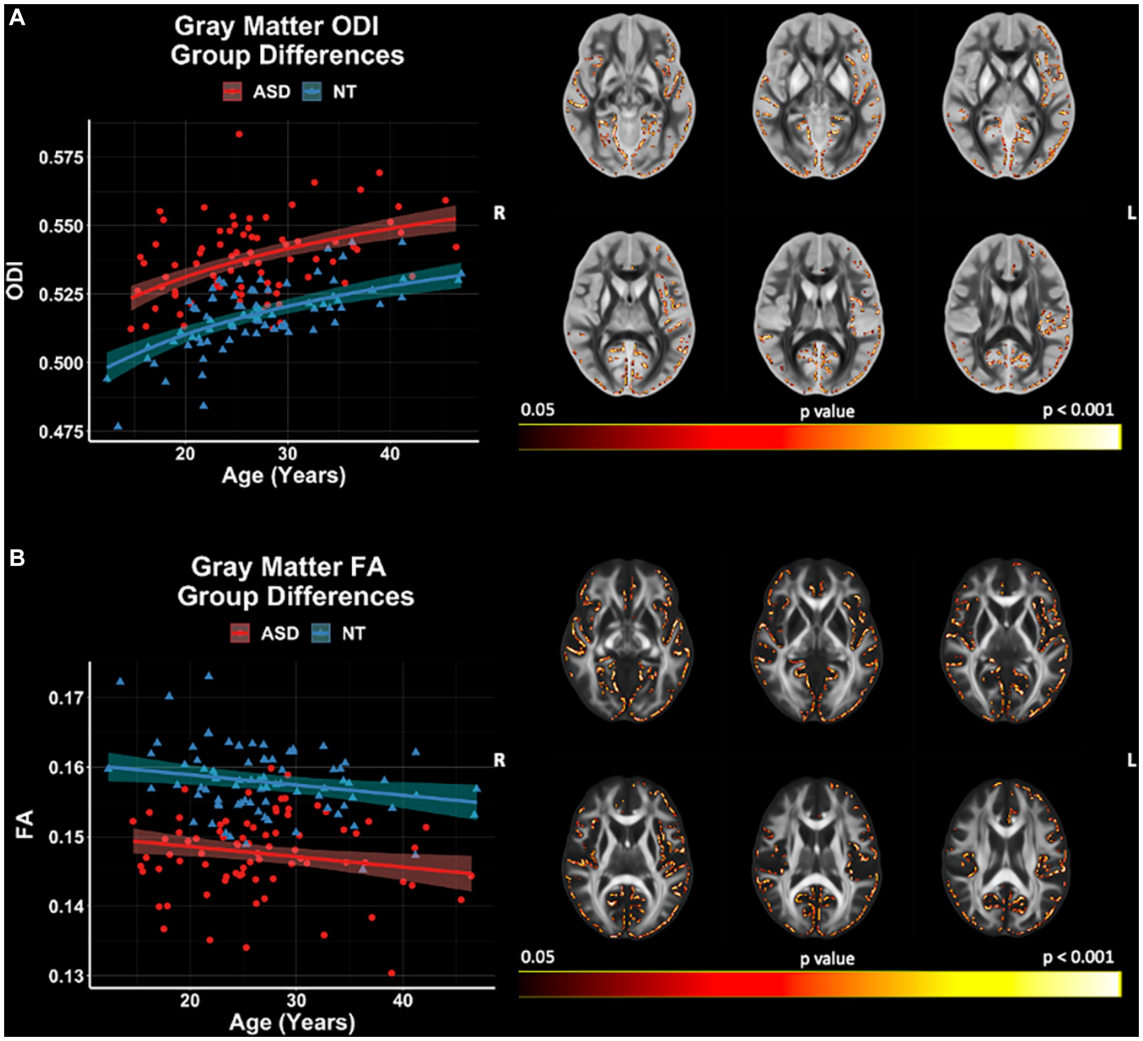
Group differences in gray matter microstructure across NODDI and DTI measures. Level of significance and neuroanatomical location of voxels from group difference model are displayed on the mean dMRI maps from all participants. Scatter points (NT = blue; ASD = red) represent the mean dMRI values of significant voxels for each measure. Trendlines show model prediction and 95% confidence intervals for group difference on dMRI measures when accounting for the effects of age. **(A)** Significant group difference of ODI. **(B)** Significant group difference of FA.

GBSS group comparisons revealed significant (*p* < 0.05, FWER-corrected) FA and ODI differences between ASD and NT groups (Figure 7). The ASD group demonstrated higher ODI, and lower FA compared to the NT group. ODI and FA were both observed to differ between the groups in the right frontal pole, frontal orbital cortex, insular cortex, lingual gyrus parahippocampal gyrus, among others. No significant group differences in GBSS were observed for measures of FICVF, MD, RD, or AD. All neuroanatomical locations of significant group differences by dMRI measure can be found in Supplementary Table S2.

dMRI measures of ODI and FA were significantly different between groups in both white matter and gray matter (Supplementary Figure S1). White matter and gray matter regions within proximity that display a significant group difference in ODI and FA include bilateral external capsules and insular cortices, bilateral posterior thalamic radiations and lingual gyri, the genu of the corpus callosum and cingulate gyri, and the left hemisphere cingulum bundle and parahippocampal gyrus.

### Age-by-group interactions across white and gray matter microstructure

Age-by-group interactions across white and gray matter microstructure were non-significant after corrections for multiple comparisons. However, several relationships were observed at *p* < 0.01, uncorrected. In the TBSS analysis, uncorrected (*p* < 0.01) age by group interactions were observed for AD such that the NT group showed AD decreases with age, whereas the AD in the ASD group slightly increased with age (Supplementary Figure S2). For GBSS, uncorrected (*p* < 0.01) age by group interactions were observed for measures of ODI, FA, MD, and AD (Supplementary Figure S3). The NT group showed increased ODI with age, whereas ODI in the ASD group slightly decreased with age. FA in gray matter decreased with age in the NT group but increased with age in the ASD group. MD and AD were seen to decrease with age in the NT group, and slightly increase and remain unchanged with age in the ASD group, respectively.

### Associations of white and gray matter microstructure with ADOS- CSS and SRS

**Figure 8:**
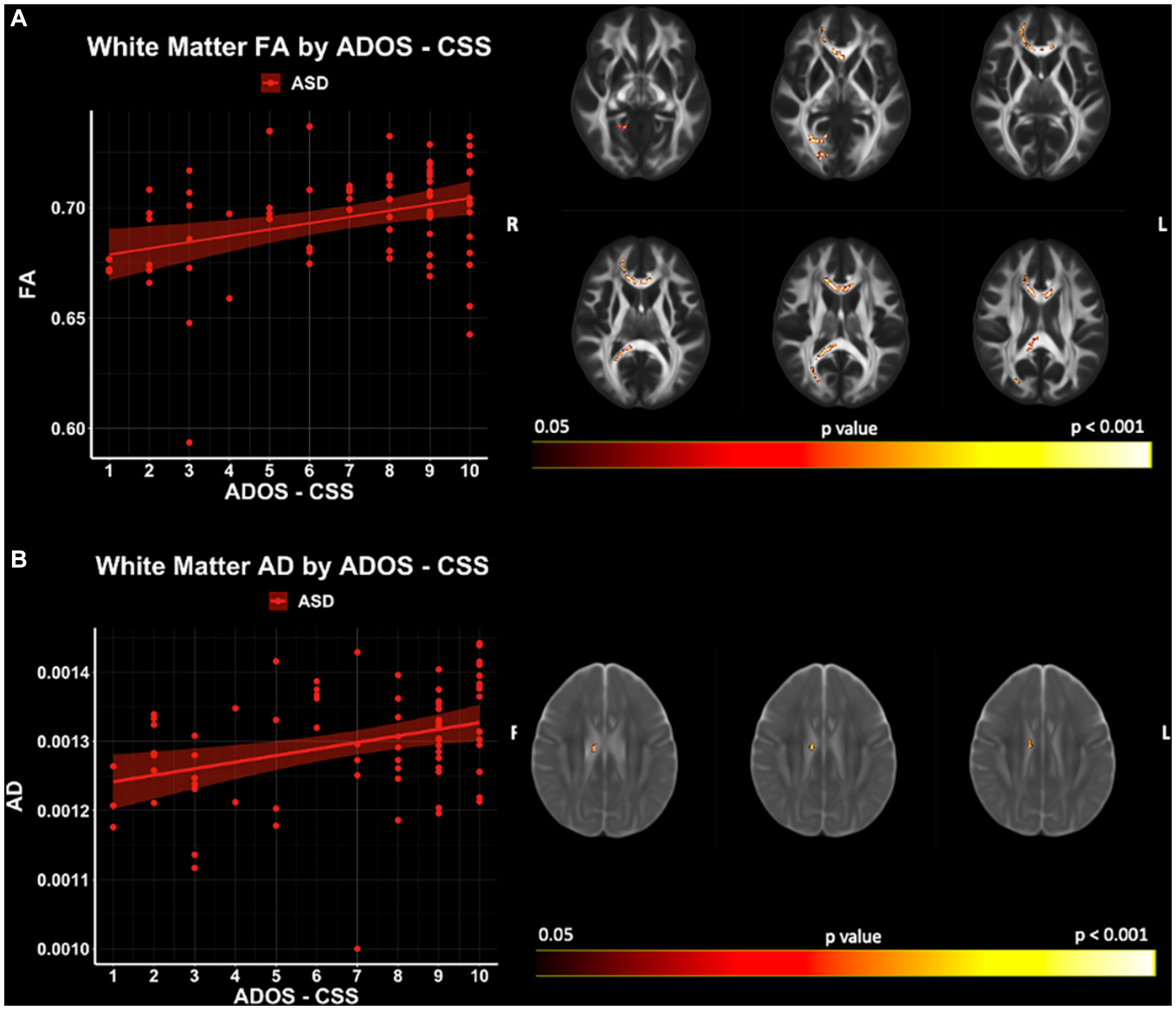
White matter microstructure relationships of **(A)** FA and **(B)** AD with ADOS- CSS in ASD. Level of significance and neuroanatomical location of significant voxels are displayed on the dMRI maps. Scatter plots represent mean dMRI values of significant voxels for each measure. Trendlines show model prediction and 95% confidence intervals for relationship between dMRI measures and ADOS-CSS when accounting for the effects of age and IQ.

Significant relationships between ADOS-CSS and white matter microstructure (accounting for the effects of age and IQ) were observed with TBSS for FA and AD within the ASD cohort (*p* < 0.05; FWER-corrected) (Figure 8; Supplementary Table S2). FA and AD were found to be positively related to ADOS-CSS in the genu and body of the corpus callosum. No significant relationships between ADOS-CSS and GBSS gray matter measures were observed in this sample. Furthermore, no significant relationships between SRS and microstructure measures in either white matter or gray matter were observed in this sample.

## Discussion

Autism spectrum disorders are thought to arise from atypical brain development leading to cascading and long-term differences in the structural and functional organization of the brain. However, little is known regarding the microstructural brain changes occurring in autistic individuals within the period from adolescence-into-adulthood, and how these neurological differences may subserve the autistic phenotype in this age-range. As such, this study aimed to examine age-related differences across whole brain white matter and gray matter microstructure between autistic and non-autistic individuals. Using TBSS and GBSS, we report widespread group differences across white and gray matter microstructure, with white matter microstructure related to a clinical score of autism severity (ADOS-CSS). Our findings provide new insights into group differences in white and gray matter organization in ASD, with evidence for a role of white matter tract organization in the level of autistic symptoms in this age-range.

Our white matter DTI findings complement the literature, with widespread decreases in FA, and increases in MD and RD (Alexander et al., 2007a; Groen et al., 2011; Kleinhans et al., 2012; Travers et al., 2015). While previous studies of ASD often report null findings of white matter differences in AD (Lee et al., 2007; Alexander et al., 2007a; Ameis et al., 2011; Jeong et al., 2011; Travers et al., 2012), reduced AD in autistic children was found in a TBSS study within prefrontal and thalamic white matter (Barnea-Goraly et al., 2010). Further, an ASD study including children-to-adult age individuals showed similar reductions in AD in ASD compared to the NT group in widespread white matter regions including the dorsolateral prefrontal cortex, superior longitudinal fasciculus, corpus callosum, among others (Noriuchi et al., 2010). These findings of reduced AD may reflect decreased axon diameter, decreased fiber density, or lack of fiber coherence. Reductions in white matter AD in the ASD group were noted in the current study within areas of the internal capsules and white matter of the brain stem. Similar findings of lower AD were also reported in autistic children compared to NT children 5-to-14 years old in the superior cerebellar peduncle (Hanaie et al., 2013).

Still, only a few studies to date have applied NODDI to investigate differential microstructural characteristics of the brain in autistic adolescents and adults with conflicting findings (Matsuoka et al., 2020; Yasuno et al., 2020; Andica et al., 2021). For example, Andica et al. (2021) reported decreased neurite density in the autistic group compared to the non-autistic group in long-range association and commissural tracts and no differences in neurite dispersion. Similar trends of decreased neurite density in ASD have been observed in cortical gray matter, with no differences in neurite dispersion (DiPiero et al., 2022). However, Matsuoka et al. reported increased neurite dispersion in ASD in gray matter regions of the visual cortex (Matsuoka et al., 2020) whereas Yasuno et al. did not detect group differences in neurite density or dispersion across white or gray matter (Yasuno et al., 2020). In the current work, we report decreased neurite density (as indexed by reduced FICVF) in white matter and increased orientation dispersion in both white and gray matter in the autistic group compared to the NT group in many regions reported in previous work (Matsuoka et al., 2020; Andica et al., 2021). Furthermore, our findings of increased ODI in gray matter were accompanied by a decrease in FA across widespread gray matter regions including the cingulate gyrus, frontal poles, lingual gyrus, among others (see Supplementary Table S2), highlighting the potential role of NODDI in disentangling the undefined biological contributions to these microstructural differences in gray matter. As current reports are conflicting, likely due to sample heterogeneity, large scale longitudinal studies needed to apply NODDI metrics to investigate white and gray matter microstructural changes in ASD across the lifespan.

White matter microstructure connects gray matter regions allowing for highly efficient and precise temporal communication across and between brain regions. As major white matter tracts play a large role in neural communication, and in turn, behavior, autistic traits have long been postulated to result from differences in structural and functional brain connectivity (Travers et al., 2012; Ameis and Catani, 2015; Dean et al., 2016). We report relationships between white matter microstructure and measure of autism severity derived from the ADOS-CSS. Specifically, we describe positive associations between white matter microstructure FA and AD and ADOS-CSS in the genu, body, and splenium of the corpus callosum, posterior thalamic radiation, anterior corona radiata, and the middle cerebellar peduncle (Figure 8; see Supplementary Table S3). Given the large clinical and biological heterogeneity across autistic individuals, studies linking the brain’s microstructure to autistic traits have been inconsistent. For example, negative relationships have been reported between restricted and repetitive behaviors and FA in major white matter tracts in autistic children and adults (Thakkar et al., 2008; Fitzgerald et al., 2019). Although not significant, trend-level relationships have also suggested negative associations between FA and autism severity measured by the ADOS in autistic adults within the body of the corpus callosum (Kleinhans et al., 2012). In another study of autistic adults, no significant correlations between white matter microstructure and clinical severity measured by the ADI-R and ADOS were found (Catani et al., 2016). Although gray matter regions in the current sample were not related to autistic traits measured by the SRS or ADOS-CSS, a gray matter study of younger autistic males found ADOS-CSS to be negatively related to ODI, and positively related to MD, RD, and AD (DiPiero et al., 2022). These inconsistent findings point to the wide heterogeneity of brain morphology in autistic individuals that change with age and warrant specific longitudinal investigations between neurological correlates of the clinical phenotypes of ASD across the lifespan.

A major goal of the current study was to investigate potential age-related differences between diagnostic groups in white and gray matter organization within the period from adolescence into adulthood. While the developmental trajectories of gray matter microstructure are not well defined, positive relationships between neurite density and myelin content and negative relationships between ODI and cortical thickness have been described in adulthood (Fukutomi et al., 2018). Developmental trajectories of white matter microstructure have been shown to follow a second order polynomial pattern peaking around mid-adulthood and rapidly decreasing in old age (Yeatman et al., 2014). Although our study did not detect significant age-related differences between diagnostic groups across white or gray matter, trend level associations were noted (see Supplementary Figures S2, S3) and may be a hallmark of a plateau in brain maturation within this developmental period. For example, a longitudinal study of white matter maturation in autistic individuals investigated age-by-group interactions on FA of the corpus callosum across different age bins (less than 10 years, 10 to 20 years, and greater than 20 years) and reported significant age-by-group interactions for FA in all subregions for individuals under 10 years of age, but no significant interactions for either of the older groups (Travers et al., 2015). The groupwise trajectories of FA curves crossed during childhood, leading to a sustained decrease in FA in the ASD group relative to NT during adolescence and young adulthood. These longitudinal results from Travers et al., 2015 suggest a differential trajectory in early childhood development of the corpus callosum microstructure in ASD that transitions into sustained group differences in adolescence and adulthood. Findings converge with the results of our current study with sustained group differences in tissue microstructure, with an absence of a significant age-by-group interaction potentially capturing structural brain differences established prior to this developmental stage that may continue to change with advanced aging; hence, our adolescent-to-adult sample may capture the period of a developmental plateau with persistent group-wise differences. This normalization trend in the developmental organization of white matter microstructure from adolescence to mid-adulthood in ASD is reported in other white matter studies (Kleinhans et al., 2012), however, more work is needed to investigate the age-related trajectories occurring in gray matter and into advanced aging.

There are a few notable limitations to the current study. First, the cross-sectional design and all male participant sample limit our ability to evaluate individual differences and may not be generalizable to autistic females. At the initial outset of this study, male participants were prioritized to decrease heterogeneity and have adequate power (Prigge et al., 2021). Additional studies of autistic females are needed to address the disparities in ASD research surrounding non-male participants to provide equitable opportunities for the development of individualized supports into adulthood. Future work to include autistic individuals with intellectual disabilities that may currently exclude them from participating in MRI studies are also needed to expand the generalizability of our results, and to aid in improving outcomes for autistic adults. Additionally, longitudinal studies are necessary for gaining a deeper understanding of brain development especially in aging as other factors, such as life experience, may influence structural and functional brain development and behavior.

To assess autism severity, clinical measures from the ADOS-2 (calibrated severity score (ADOS-CSS)) and SRS (total T-Score) were used. While the ADOS-2 is an activity-based diagnostic tool administered by trained clinicians to assess autistic symptoms during a standardized evaluation, the SRS is a parent-based questionnaire that encompasses a broader range of behaviors observed by the parent across a wider context and time frame. The complementary information provided by these two assessments strengthens the interpretability of our study (Hus et al., 2013a,b, 2014). However, it remains unclear how these severity scores and their underlying brain structures change with advancing age and how the temporal emergence of brain-behavior relationships changes across the lifespan.

Our study leverages information from both the DTI and NODDI models to investigate age relationships, group differences, and age-related differences between diagnostic groups. While both models are distinctly different models to describe the underlying dMRI signal, with DTI serving as a signal representation model and NODDI a biophysical model (Basser et al., 1994a,b; Alexander et al., 2007b; Zhang et al., 2012; Jelescu et al., 2020), use of metrics from both models allows for a more complete view of microstructural differences related to ASD. Indeed, Zhang et al., 2012, suggests that mutual use of DTI and NODDI metrics for completeness, particularly in clinical studies, may be desirable as other non-neurite sources may contribute to changes in DTI-based metrics (Zhang et al., 2012). Future work examining the relationships between DTI and NODDI as well as investigating the sensitivity of these models to underlying biological factors are needed. Lastly, although inclusion of DTI metrics allows for our findings to be interpreted in the context of previous work, DTI metrics, particularly in gray matter, may be come unstable as the diffusion environment becomes more isotropic (Lenglet, 2015) and is thus considered a limitation of our study.

In conclusion, the current study investigated white and gray matter microstructural differences between autistic and non-autistic individuals using advanced dMRI, TBSS, and GBSS. Findings revealed group differences in white and gray matter organization in brain areas involved in various cognitive, sensory, and motor functions. Furthermore, this work begins to bridge a critical gap in knowledge surrounding brain organization related to ASD from adolescence into mid-adulthood. To our knowledge, this study is the first to utilize NODDI in conjunction with the TBSS and GBSS frameworks to assess whole brain microstructural differences in ASD and supports the hypothesis that differences in both neural circuitry and cortical microstructure play an important role in ASD. Future studies are necessary to assess how these microstructural brain differences continue to change into late-adulthood, and how these structural changes may support future behavioral challenges in autistic adults. Findings from this study will help guide future longitudinal studies of ASD from early life into late-adulthood and may ultimately inform development of improved treatment options for autistic individuals based on brain-behavior relationships.

## Data availability statement

The raw data supporting the conclusions of this article will be made available by the authors, without undue reservation.

## Ethics statement

The studies involving humans were approved by the Institutional Review Boards at the University of Utah and University of Wisconsin–Madison. The studies were conducted in accordance with the local legislation and institutional requirements. Written informed consent for participation in this study was provided by the participants’ legal guardians/next of kin.

## Author contributions

MD: conceptualization, methodology, software, formal analysis, investigation, writing – original draft, writing – review and editing, visualization, and project administration. HC: formal analysis, data organization, writing – review and editing, and visualization. MP, CK, and JK: data curation, data organization, data resources, and writing – review and editing. JG-G: methodology, data curation, resources, visualization, and writing – review and editing. NA: methodology, data curation, and writing – review and editing. NL and EB: data curation, data resources, and writing – review and editing. BZ and JL: methodology, resources, data curation, writing – review and editing, supervision, and funding acquisition. AA: conceptualization, methodology, data resources, technical resources, data curation, writing – review and editing, supervision, funding acquisition, and project administration. DD: conceptualization, methodology, software, data resources, data curation, technical resources, writing – review and editing, supervision, funding acquisition, and project administration. All authors contributed to the article and approved the submitted version.
