# Supplementary material for: Tract- and gray matter- based spatial statistics show white matter and gray matter microstructural differences in autistic males
Source: Front Neurosci. 2023 Sep 27;17:1231719. doi: 10.3389/fnins.2023.1231719 (PMC10565827; doi:10.3389/fnins.2023.1231719)
Supplement: Supplementary file 1 [file Data_Sheet_2.DOCX]

Supplementary Material

**Tract- and Gray Matter- Based Spatial Statistics Show White Matter and Gray Matter Microstructural Differences in Autistic Males**

**Authors**: Marissa DiPiero^1,2^, Hassan Cordash^2^, Molly B. Prigge^3^, Carolyn K. King^3^, Jubel Morgan^3^, Jose Guerrero-Gonzalez^2^, Nagesh Adluru^2,4^, Jace B. King^3^, Nicholas Lange^5^, Erin D. Bigler^3,6,7,8,9^, Brandon A. Zielinski^3,6,10,11,12^, Andrew Alexander^2,13,14^, Janet E. Lainhart^2,13^, and Douglas C. Dean III^2,14,15^

^1^Neuroscience Training Program, University of Wisconsin-Madison, Madison, WI

^2^Waisman Center, University of Wisconsin-Madison, Madison, WI

^3^Department of Radiology and Imaging Sciences, University of Utah, Salt Lake City, UT

^4^ Department of Radiology, University of Wisconsin-Madison, Madison, WI

^5^Department of Psychiatry, Harvard School of Medicine, Boston, MA

^6^Department of Neurology, University of Utah, Salt Lake City, UT

^7^Department of Psychiatry, University of Utah, Salt Lake City, UT

^8^Psychology and Neuroscience Center, Brigham Young University, Provo, UT

^9^Neurology, University of California-Davis, Davis, CA

^10^Department of Pediatrics, University of Utah, Salt Lake City, UT

^11^Departments of Pediatrics and Neurology, University of Florida, Gainesville, FL

^12^ McKnight Brain Institute, University of Florida, Gainesville, FL

^13^Department of Psychiatry, University of Wisconsin-Madison, Madison, WI

^14^Department of Medical Physics, University of Wisconsin-Madison, Madison, WI

^15^Department of Pediatrics, University of Wisconsin-Madison, Madison, WI

*Address Correspondence to:* Douglas C. Dean III

Waisman Center

University of Wisconsin–Madison

Madison, WI, USA, 53705

Tel. # +1 608.262.6706

Email: [deaniii@wisc.edu](mailto:deaniii@wisc.edu)

**
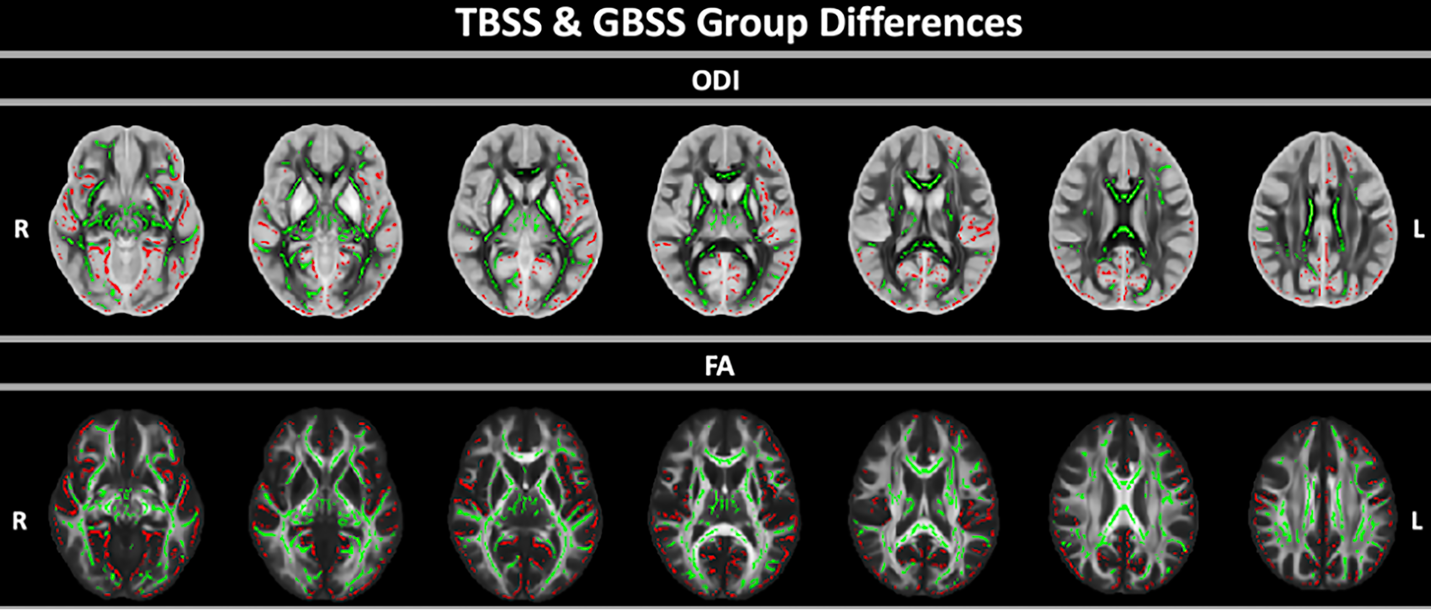
**

**Supplement Figure 1:** TBSS and GBSS Group Differences in ODI and AD. Green voxels represent white matter voxels displaying a significant group difference. Red voxels represent gray matter voxels displaying a significant group difference.


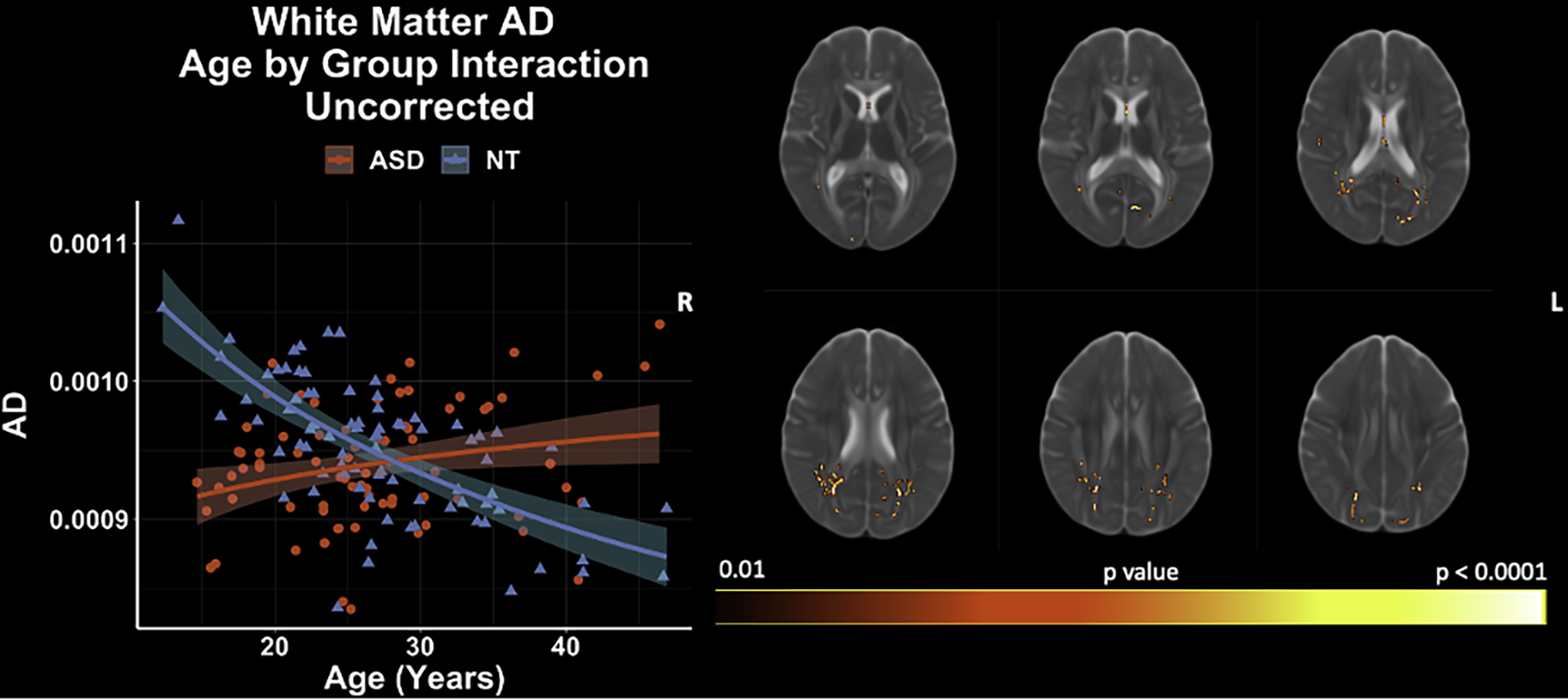


**Supplement Figure 2:** Uncorrected Age-by-group Interactions on white matter in TBSS analysis.


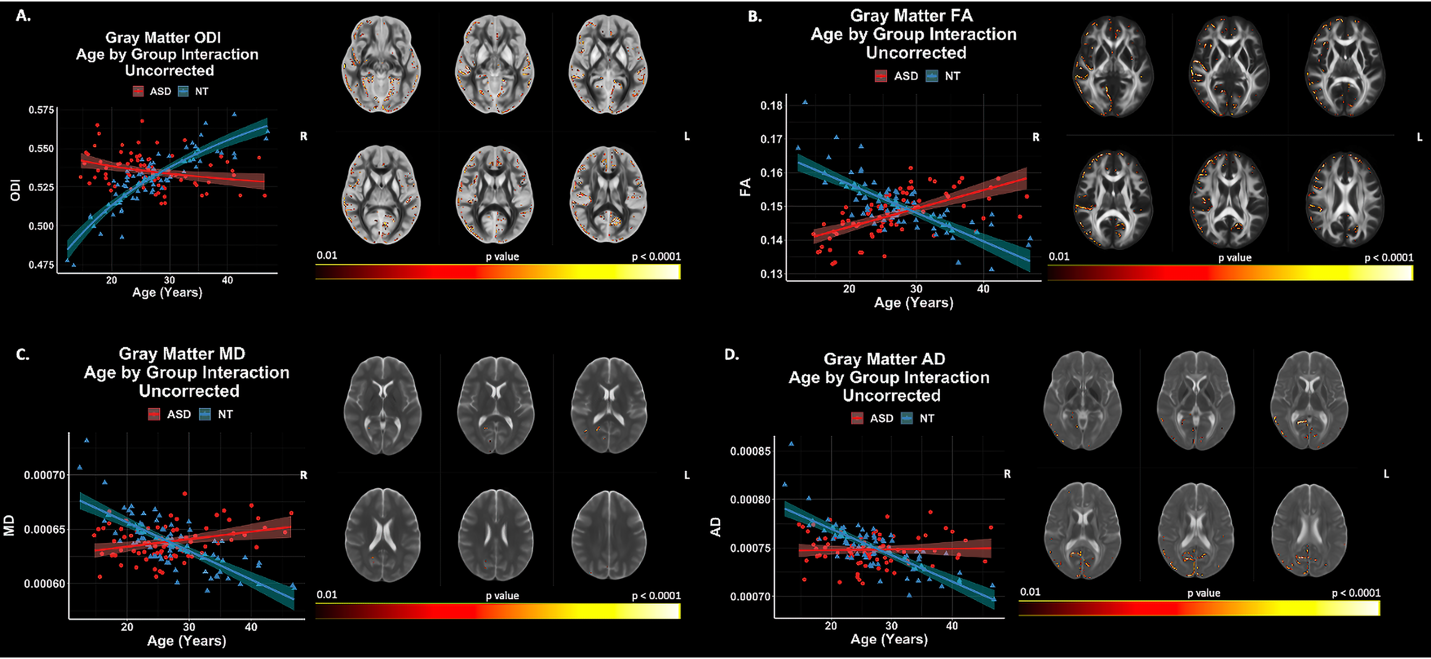


**Supplement Figure 3:** Uncorrected age-by-group interactions on gray matter in GBSS analysis.

| **Supplement Table 1: Neuroanatomical Locations of Significant Group Differences in White Matter** | | | | | | |
| --- | --- | --- | --- | --- | --- | --- |
| **Hemisphere** | **DWI Measures** | | | | | |
|  | **FICVF** | **ODI** | **FA** | **MD** | **RD** | **AD** |
| **Bilateral** | – Anterior corona radiata  – Body of corpus callosum  – External capsule  – Fornix  – Genu of corpus callosum  – Posterior corona radiata  – Posterior thalamic radiation  – Retrolenticular part of internal capsule  – Sagittal stratum  – Superior corona radiata  – Superior longitudinal fasciculus  – Uncinate fasciculus | – Anterior corona radiata  – Anterior limb of internal capsule  – Body of corpus callosum  – Cerebellar penducle  – Cerebral peduncle  – Corticospinal tract  – External capsule  – Fornix  – Genu of corpus callosum  – Inferior cerebellar peduncle  – Medial leminiscus  – Pontine crossing tract  – Posterior limb of internal capsule  – Posterior thalamic radiation  – Retrolenticular part of internal capsule  – Sagittal stratum  – Splenium of corpus callosum  – Superior cerebellar peduncle  – Superior corona radiata  – Superior longitudinal fasciculus | – Anterior limb of internal capsule  – Body of corpus callosum  – External capsule  – Fornix  – Genu of corpus callosum  – Posterior corona radiata  – Posterior limb of internal capsule  – Retrolenticular part of internal capsule  – Sagittal stratum  – Splenium of corpus callosum  – Superior longitudinal fasciculus | – | – Anterior corona radiata  – Body of corpus callosum  – External capsule  – Genu of corpus callosum  – Posterior limb of internal capsule  – Retrolenticular part of internal capsule  – Splenium of corpus callosum  – Superior corona radiata  – Superior longitudinal fasciculus | – Cerebral peduncle  – Corticospinal Tract  – Inferior cerebellar peduncle  – Posterior limb of internal capsule  – Superior cerebellar peduncle |
| **Left** | – Anterior limb of internal capsule  – Cingulum (hippocampus)  – Posterior thalamic radiation  – Superior fronto-occipital fasciculus |  | – Cingulum (hippocampus) | – | – Posterior thalamic radiation | – Anterior limb of internal capsule  – Medial leminiscus  –Retrolenticular part of internal capsule |
| **Right** | – Anterior limb of internal capsule  – Cingulum (hippocampus)  – Posterior thalamic radiation  – Superior fronto-occipital fasciculus  – Cingulum (cinguluate gyrus)  – Splenium of corpus callosum  – Tapetum |  | – Superior corona radiata  – Anterior corona radiata  – Cerebral peduncle | – Anterior corona radiata  – Genu of corpus callosum | – Posterior corona radiata  – Sagittal stratum | – Middle cerebellar peduncle |

| **Supplement Table 2: Neuroanatomical Locations of Significant Group Differences in Gray Matter** | | | | | | | |
| --- | --- | --- | --- | --- | --- | --- | --- |
| **Hemisphere** | **DWI Measures** | | | | | | |
|  | **FICVF** | **ODI** | **FA** | **MD** | **RD** | **AD** |  |
| **Bilateral** | – | – Angular gyrus  – Cingulate gyrus, posterior division  – Cuneal cortex  – Frontal orbital cortex  – Frontal pole  – Intracalcarinal cortex  – Lateral occipital cortex, superior division  – Lingual gyrus  – Middle temporal gyrus, posterior division  – Occipital fusiform gyrus  – Parahippocampal gyrus  division  – Parietal operculum cortex  – Planum pole  – Precuneus cortex  – Superior temporal gyrus, anterior division  – Supramarginal gyrus, posterior division  – Temporal pole | – Central opercular cortex  – Cingulate gyrus, posterior division  – Frontal orbital cortex  – Frontal pole  – Inferior frontal gyrus, pars opercularis  – Insular cortex  – Lateral occipital cortex, superior division  – Lingual gyrus  – Middle temporal gyrus, posterior division  – Middle temporal gyrus, temporo occipital part  –Occipital Pole  – Paracingulate gyrus  – Parahippocampal gyrus  – Precentral gyrus  – Precuneus cortex  – Subcallosal cortex  – Temporal fusiform cortex, posterior division  – Temporal pole | – | – | – |  |
| **Left** | – | – Temporal occipital fusiform  – Occipital pole  – Lateral occipital cortex, inferior division  – Central opercular cortex  – Insular cortex  – Central opercular cortex  – Frontal operculum cortex  – Inferior frontal gyrus, pars triangularis  – Cingulate gyrus, anterior division  – Postcentral gyrus  – Paracingulate gyrus  – Precentral gyrus  – Middle frontal gyrus  – Paracingulate gyrus  – Superior frontal division  – Supramarginal gyrus, anterior division | – Cingulate gyrus, anterior division  – Inferior frontal gyrus, pars triangularis  – Lateral occipital cortex, inferior division  – Middle frontal gyrus  – Occipital fusiform gyrus, superior frontal division  – Temporal occipital fusiform cortex  – Middle temporal gyrus, anterior division  – Middle temporal gyrus, posterior division | – | – | – |  |
| **Right** | – | – | – | – | – | – |  |

| **Supplement Table 3: Neuroanatomical Locations of Significant ADOS Relationships in White Matter** | | | | | | |
| --- | --- | --- | --- | --- | --- | --- |
| **Hemisphere** | **DWI Measures** | | | | | |
|  | **FICVF** | **ODI** | **FA** | **MD** | **RD** | **AD** |
| **Bilateral** | – | – | – Genu of corpus callosum  – Body of corpus callosum  – Splenium of corpus callosum  – Middle cerebellar peduncle | – | – | – |
| **Left** | – | – | – | – | – | – |
| **Right** | – | – | – Anterior corona radiata  – Posterior thalamic radiation | – | – | – Body of corpus callosum |
